# Key Regulatory Role of Dermal Fibroblasts in Pigmentation as Demonstrated Using a Reconstructed Skin Model: Impact of Photo-Aging

**DOI:** 10.1371/journal.pone.0114182

**Published:** 2014-12-09

**Authors:** Christine Duval, Catherine Cohen, Corinne Chagnoleau, Virginie Flouret, Emilie Bourreau, Françoise Bernerd

**Affiliations:** L'Oréal Research & Innovation, Aulnay-sous-Bois, France; University of Tennessee, United States of America

## Abstract

To study cutaneous pigmentation in a physiological context, we have previously developed a functional pigmented reconstructed skin model composed of a melanocyte-containing epidermis grown on a dermal equivalent comprising living fibroblasts. The present studies, using the same model, aimed to demonstrate that dermal fibroblasts influence skin pigmentation up to the macroscopic level. The proof of principle was performed with pigmented skins differing only in the fibroblast component. First, the *in vitro* system was reconstructed with or without fibroblasts in order to test the global influence of the presence of this cell type. We then assessed the impact of the origin of the fibroblast strain on the degree of pigmentation using fetal versus adult fibroblasts. In both experiments, impressive variation in skin pigmentation at the macroscopic level was observed and confirmed by quantitative parameters related to skin color, melanin content and melanocyte numbers. These data confirmed the responsiveness of the model and demonstrated that dermal fibroblasts do indeed impact the degree of skin pigmentation. We then hypothesized that a physiological state associated with pigmentary alterations such as photo-aging could be linked to dermal fibroblasts modifications that accumulate over time. Pigmentation of skin reconstructed using young unexposed fibroblasts (n = 3) was compared to that of tissues containing natural photo-aged fibroblasts (n = 3) which express a senescent phenotype. A stimulation of pigmentation in the presence of the natural photo-aged fibroblasts was revealed by a significant increase in the skin color (decrease in Luminance) and an increase in both epidermal melanin content and melanogenic gene expression, thus confirming our hypothesis. Altogether, these data demonstrate that the level of pigmentation of the skin model is influenced by dermal fibroblasts and that natural photo-aged fibroblasts can contribute to the hyperpigmentation that is associated with photo-aging.

## Introduction

In human skin, melanocytes that produce the melanin pigment accounting for the tegument color, lie at the dermal epidermal junction (DEJ), the boundary structure between the two major skin compartments, the epidermis and dermis. Melanocytes in the basal layer of epidermis produce various amounts and types of melanins (eu/pheomelanins) within specific organelles called melanosomes that are transferred to neighboring epidermal keratinocytes. During the keratinocyte differentiation program, melanosomes are more or less degraded depending on the skin type, leading to melanin dust in fair skin or unaltered melanosomes all the way up to the outermost epidermal layers in black skin. The large variety of constitutive colors of human skin arises from these complex and gradual processes.

Precise molecular and cellular events underlying regulation and dysregulation of pigmentation are partially known. The melanin biosynthetic pathway leading to polymerized end-products starts with the amino acid L-tyrosine and the key regulator enzyme tyrosinase. For eumelanins which give rise to brown or black pigment (comprising DHICA-melanin and DHI-melanin), TRP-1 (tyrosinase related protein-1) and TRP-2 (tyrosinase related protein-2, or DCT for DOPA-chrome tautomerase) are involved. The presence of L-cystein is required for the synthesis of the more reddish pheomelanin pigments. The tyrosinase, TRP-1 and TRP-2 genes involved in melanogenesis are all under the control of the master transcription factor MITF (Microphthalmia Transcription Factor). Important regulators of melanogenesis are the POMC derived peptides (αMSH, ACTH) found in both the epidermis and the dermis [1], [2]. They are produced by various cell types including keratinocytes, melanocytes, fibroblasts and endothelial cells. Some signals (UV, IL1, cAMP) are known to upregulate POMC peptides as well as αMSH receptor MC1R in a paracrine, autocrine or intracrine manner [3]. Melanin synthesis is regulated by additional locally produced factors including growth factors, inflammatory mediators, neurotransmitters, neuropeptides and hormones (namely the oestrogenic and glucocorticoid systems) [4], [5]. Organized similarly to the hypothalamo-pituitary-adrenal axis, the cutaneous corticotropin-releasing factor (CRF)/urocortin signaling system, comprising CRF, POMC, αMSH and ACTH, can participate to the regulation of melanocyte homeostasis. CRF signaling through cognate CRF receptors increases levels of cAMP, inositol triphosphate (IP3) or Ca2+, activates protein kinases A and C pathways and ultimately stimulates melanocyte proliferation, differentiation and pigment production [6]. As proposed by Slominski et al [7], other positive regulators of pigmentation are melanin precursors such as L-Tyrosine and L-DOPA, which besides being major substrates of melanogenesis, can promote the proper folding and activity of tyrosinase and also the formation and maturation of melanosomes.

In the skin, melanocytes are in tight contact with neighboring keratinocytes and a large number of studies have highlighted the role of keratinocytes in the control of skin pigmentation [8]–[10]. For example, keratinocytes contribute to transient UV-induced melanogenesis (tanning) by releasing numerous pro-pigmenting paracrine growth factors such as αMSH, Endothelin-1 (ET-1), Stem cell factor (SCF) and various cytokines.

However, increasing evidence has underlined the contribution of dermal components in the regulation of pigmentation. In the mid 1990's, extracellular matrix proteins (ECM) were shown to regulate melanocyte proliferation, apoptosis resistance and melanogenic activity [11]–[13]. More recently, dermal fibroblasts were demonstrated to exert a regulatory role on pigmentation through the secretion of soluble factors. Regarding constitutive pigmentation, Dikkopf-1(DKK-1) produced by fibroblasts in the palms and soles is thought to be responsible for the lighter color of these body sites via its suppressive effect on melanocyte growth and activity as well as on melanosome transfer [14], [15]. In contrast, the pro-pigmenting effect of neuregulin-1 (NGR-1) secreted by dark skin fibroblasts suggests that this particular signaling molecule plays a role in determining highly pigmented skin color type [16]. In addition, in various congenital hyperpigmented disorders such as systemic scleroderma, dermatofibroma, café-au-lait macules of neurofibromatosis, and generalized progressive dyschromatosis, an increase in the production of growth factors known to be melanogenic has been reported in the dermal compartment; these factors include SCF, hepatocyte growth factor (HGF) or keratinocyte growth factor (KGF) [17]–[21]. Tight reciprocal interactions between epidermal and mesenchymal compartments may also play a role in melanocyte homeostasis. It has recently been shown that laminin 332 a major component of the epidermal basement membrane contributes to melanin production by regulating L-tyrosine uptake [22]. Growth factor/cytokine regulatory loops also exist between the two cellular compartments and contribute to the regulation of pigmentation. For example, keratinocyte-produced cytokines, such as interleukin 1 alpha (IL1α) or tumor necrosis factor alpha (TNFα), may stimulate fibroblasts which in turn release melanocyte-stimulating factors such as HGF or SCF [23]–[25].

All of these converging elements increasingly underline the role of dermal fibroblasts in regulating constitutive pigmentation and in the development of pigmentary disorders. We therefore hypothesized that physiological alterations of fibroblasts over time, notably photo-aging, might influence pigmentary status. Skin photo-aging induced by chronic solar UV exposure is associated with pigmentation alterations and the formation of actinic (or senilis) lentigines, a hallmark of photo-aged skin [26]–[32]. Recently, Kovacs et al [33] described an increase in immunodetection of melanogenic cytokines in the upper dermis of skin with senilis lentigines and suggested a fibroblast contribution to this hyperpigmentation.

Most of the *in vitro* studies aimed at demonstrating the role of dermal fibroblastic components in the regulation of pigmentation have been performed in 2D cell cultures (melanocytes in the presence of secreted fibroblast factors or melanocyte/keratinocyte co-cultures, for example). A few experiments attempted to set up a 3D approach using a fibroblast monolayer cultured underneath a pigmented reconstructed epidermis. These systems do not recapitulate the physiological matrix environment for dermal fibroblasts nor the tissue architecture necessary for the three cell types interaction. Working with an appropriate model is therefore one of the keys to clarify the link between skin pigmentation and fibroblast activity/phenotype. An *in vitro* full-thickness pigmented skin model, which allows physiological interactions to take place between the melanocyte-containing epidermis and the dermal compartment including living fibroblasts, is thus needed to study the influence of dermal fibroblast on pigmentation. Commonly available 3D skin models do not comprise both a pigmented epidermis and a fibroblast-populated dermal equivalent. To overcome the absence of fibroblasts in 3D pigmented models, several teams have developed pigmented skin models comprising a living dermal equivalent on various dermal substrates (dead de-epidermized dermis, glycoaminoglycan-collagen sponge, fibroblast-populated collagen gel) [34]–[39]. However these models reported drawbacks especially the limited survival or the lack of functionality of normal melanocytes. We recently developed a more satisfactory pigmented skin model comprising a melanocyte-containing epidermis cultured on a living fibroblasts embedded-dermal equivalent [40]. Using various culture conditions and the addition of KGF during the air-liquid interface culture phase, we succeeded in correctly integrating melanocytes at the basal layer of the epidermis and in inducing a physiological differentiation of the cells as revealed by the melanin production (constitutive pigmentation). We tested the ability of the system to integrate melanocytes isolated from more or less pigmented skins and could reproduce various pigmentation phenotypes. Finally, the responsiveness of the pigmentary system was verified by stimulation of pigmentation with known propigmenting references, αMSH and forskolin, or its reduction with a depigmenting agent. We thus showed, in 2012, that this pigmented skin model reproduces the 3D architecture of the melanocyte environment, allows physiological interactions between melanocytes, keratinocytes and fibroblasts, and is functional in terms of constitutive or inducible pigmentation. Since that time, we have extensively reproduced this model using multiple melanocyte, keratinocyte and fibroblast strains and have proven the robustness of the model. The present study aimed at providing evidence of the pigmentary responsiveness of this skin model when it is submitted to a variable fibroblastic component. This model is a unique *in vitro* tool for demonstrating, from the microscopic to the macroscopic level, the key role of dermal fibroblasts in the regulation of pigmentation. To that end, two types of experiments were performed, giving rise to the proof of principle. Firstly, the global impact of dermal fibroblasts on pigmentation was examined using skins reconstructed in the presence or absence of dermal fibroblasts. Skin color, melanin content and melanocytes number were assessed in the standard skin model and compared to the skin model reconstructed on acellular dermal-equivalent (DE). In a second approach, we addressed the question of the influence of the origin/phenotype of dermal fibroblasts on pigmentation. Adult or fetal fibroblasts known to deeply differ particularly in wound healing process [41], [42], were integrated in the DE and the same pigmentation end-points were measured. Finally, we hypothesized that physiological modifications of dermal fibroblasts acquired over time could participate in modifications of skin pigmentation. Since skin photo-aging has been clinically associated with alterations of the dermal structure [43], [44] and pigmentary modifications [26]–[28], [31], we were interested to study the influence of fibroblasts from naturally photo-aged skins in the 3D model pigmentation. Hence, we compared pigmentation in a reconstructed skin model containing either fibroblasts isolated from young photo-protected adult skin or fibroblasts obtained from naturally photo-aged skins.

## Materials and Methods

### Ethics Statement

Normal human skin was obtained from surgical residues after written informed consent from the donors (or from their legal guardians for donor under the age of 18) according to the principles expressed in the Declaration of Helsinki and in article L.1243-4 of the French Public Health Code. Given its special nature, surgical residue is subject to specific legislation included in the French Code of Public Health (anonymity, gratuity, sanitary/safety rules…). This legislation does not require prior authorization by an ethics committee for sampling or use of surgical waste.

### Cell culture

Keratinocytes: Epidermal normal human keratinocytes (NHK) were isolated from adult Caucasian breast skin after plastic surgery procedures, cultured as described [45] on a feeder layer of Swiss 3T3 fibroblasts and used at passage 2 for skin reconstruction.

Melanocytes: Normal human melanocytes (NHM) were isolated from foreskin of two young moderately pigmented Caucasian donors, and amplified in a defined melanocyte culture medium M2 (Promocell, Heidelberg, Germany) containing no phorbol ester and used at passage 4–6.

Fibroblasts: Three different strains of normal human dermal fibroblasts from young adult Caucasian donors (16, 19 and 21 years old) were obtained from mammary skin coming from plastic surgery procedures. Fibroblasts were isolated using the skin explant method and amplified in Dulbecco's modified Eagle's medium (DMEM) +10% fetal calf serum (FCS). Fetal fibroblasts (GM10) were obtained from Coriell Institute (Camden, NJ, USA.) and cultured in DMED +10% FCS. Three different strains of fibroblasts isolated from facial skin of 71, 73 and 77 year-old Caucasian donors (naturally photo-aged fibroblasts, PAF) were obtained from Tebu-bio (Le Perray en Yvelines, France) and cultured in DMEM +10% FCS.

In the standard model, adult young fibroblasts were used at passage 8. For comparative studies, young adult and fetal fibroblasts were used at the same passage 8. For comparative studies between young adult and natural photo-aged fibroblasts, all the strains were used at passage 4 due to the low replicative potential of photo-aged cells.

### Skin reconstruction

NHM, NHK and fibroblasts were amplified separately in their respective growth medium. The dermal equivalent was obtained after contraction at 37°C during 4 days of a mixture of bovine type I collagen and fibroblasts (10^6^ cells for 7 ml) as previously described [46], [47]. NHK and NHM were co-seeded at a concentration of 33000 cells/cm^2^ each inside a 1.5 cm^2^ steel ring placed on top of the contracted dermal equivalent as previously described [40]. The culture was then kept immersed for 7 days allowing cells to form a monolayer. The culture was raised to the air-liquid interface (emersion phase) and kept at least for 1 week to allow the keratinocytes to stratify and differentiate. The culture medium used during the first 4 days of immersion phase was composed of Minimal Essential Medium (Invitrogen, Cergy-Pontoise, France), 10% fetal calf serum (Sigma, Saint Louis, USA), 10 ng/ml EGF (Becton Dickinson, Bedford, MA, USA), 10^−10 ^M Cholera toxin (Biomol, Plymouth, MA, USA), 0.4 µg/ml hydrocortisone (Sigma, Saint Louis, MO, USA), and supplemented with M2 melanocyte growth factors (0.625 ml/l). The culture medium containing KGF (10 ng/ml, R&D systems, Minneapolis, MN, USA) instead of EGF was used for the remaining culture period, i.e. from day 4 of the immersion phase until 11 days (for the experiments with acellular dermis) or 14 days after emersion (for the other experiments)

For comparative studies in which only the dermal fibroblast component varied, the same strains of NHK and NHM were used.

### Obtention of a dermal equivalent without fibroblasts

After contraction, the dermal equivalent was subjected to osmotic shock in water during 2 hours at room temperature. Three water rinsings were performed to get rid of all the fibroblasts as confirmed by CD13 immunohistochemistry. The acellular dermal equivalents were kept in PBS+ at +4°C for 4 to 6 weeks until they were used for skin reconstruction. All the other steps, from the seeding of NHK and NHM and onwards, were identical to those described above.

### Analysis of fibroblasts in monolayer

For the comparative study between young adult photo-protected and natural photo-aged fibroblasts, phenotypic characteristics were assessed during the amplification phase. Morphological examination was performed using bright field microscopy. Senescence-associated β-galactosidase (SA-β-gal) staining was performed according to the method of Serrano et al. [48]. Fibroblast population doubling time was estimated, for each strain, by counting the number of seeded fibroblasts and the number of harvested fibroblasts after 13 days of culture. The time required for a two-fold increase of the population (Population Doubling Time) was calculated for three independent experiments.

### Colorimetric measurements

A Mercury 2000 spectrocolorimeter (Datacolor, Montreuil, France) was used to measure the Luminance (L*) of the pigmented reconstructed skin samples (3 to 6 samples for each experiment), in the standard CIE L*a*b* color space [49]. The lower the L* value, the darker the color of the skin sample.

### Histology and immunostaining of melanocytes on skin sections

Reconstructed skin samples were fixed in 10% neutral formalin and treated for classical histology. 5 µm paraffin sections were stained with hematoxylin, eosin and saffron or colored by Fontana Masson (FM) staining for melanin detection.

For immunolabeling, samples were embedded in Tissue Tek (Miles, MT, USA) and frozen in liquid nitrogen. 5 µm cryosections were post-fixed in methanol at −20°C. To detect proteins involved in melanogenesis, primary monoclonal antibodies (MoAb), Ta99 specific to human TRP-1 (Signet, Dedham, MA, USA) and T311 specific to human Tyrosinase (Novocastra, Newcastle, UK) were used. For fluorescent immunochemistry, Ta99 (dilution 1/50) which recognizes TRP-1 was applied to cryosections for 30 min at room temperature (RT). Then Alexa 488 (dilution 1/500) or Alexa 568 (dilution 1/200) coupled secondary antibody (Molecular Probes, Eugene, OR, USA) was applied for 30 min at RT. Nuclear counterstaining was performed with propidium iodide (PI, dilution 1/200, Sigma-Aldrich, Saint Quentin Fallavier, France) or with Hoechst dye (Invitrogen, Cergy-Pontoise, France). For the enzymatic detection of tyrosinase on cryosections by T311 (1/10), biotinylated substrate specific to the secondary antibody was applied overnight at 4°C then revealed using streptavidin-biotin peroxidase complex (kit ABC, Vectastain, Burlingame, CA, USA) and 3-amino-9-ethylcarbazole (AEC) (Dako, Les Ulis, France) as enzymatic substrate. Counterstaining was performed with Papanicolaou Blue. AEC revealed slides were mounted with immuno-mount (Thermo, Pittsburgh, PA, USA).

For fluorescent staining of fibroblasts, cryosections were fixed in acetone for 5 min at 4°C, then incubated with WM15 MoAb specific to CD13 antigen (dilution 1/10; AbDSerotec, Düsseldorf, Germany) for 60 min at RT, then with Alexa 488 coupled secondary antibody for 60 min at RT (dilution 1/500; Molecular Probes). Finally, nuclear counterstaining was performed with Hoechst dye (dilution 1/2500; Invitrogen) or PI (dilution 1/200, Sigma-Aldrich).

For fluorescent co-staining of fibroblasts and melanocytes on cryosections, fibroblasts were firstly labeled with WM15 MoAb specific to CD13 antigen (dilution 1/10; AbDSerotec) which was applied for 30 min at RT. Alexa 488 coupled secondary antibody (dilution 1/150; Molecular Probes) was then applied for 30 min at RT. Fixation with methanol was performed before labeling melanocytes with Ta99 specific to hTRP-1 (dilution 1/50) and Alexa 568 coupled secondary antibody (dilution 1/200; Molecular Probes), both applied for 30 min at RT. Finally, nuclear counterstaining was performed with Hoechst dye (dilution 1/2500; Invitrogen).

Negative controls were performed by omitting primary antibodies.

### Morphology, distribution and number of melanocytes on epidermal sheets

In order to have direct access to the melanocytes residing at the basal layer for further analysis, the epidermis was separated from the dermis using forceps. The DOPA reaction was performed directly on the epidermal sheets to visualize melanocyte morphology and distribution. Also, a specific melanocyte staining using TRP1 was performed and combined with image analysis to quantify the melanocyte population.

For fluorescent TRP1 staining of melanocytes on epidermal sheets, samples were first fixed with methanol at −20°C for 10 min. Primary monoclonal antibody (MoAb) TA99 specific to TRP-1 (Signet) was applied (dilution 1/100) for 60 min at RT. Then Alexa 488 coupled secondary antibody (Molecular Probes) was applied (dilution 1/300) for 60 min at RT. Fluorescent scans of the TRP-1-stained sheets were carried out using a Hamamatsu scanner (Hamamatsu Photonics, Massy, France). Using image analysis software Histolab (Microvision instruments, Evry, France), the number of melanocytes was counted. Results were expressed as the number of melanocytes per mm^2^ of epidermal surface.

### Melanin content

**Figure 1:**
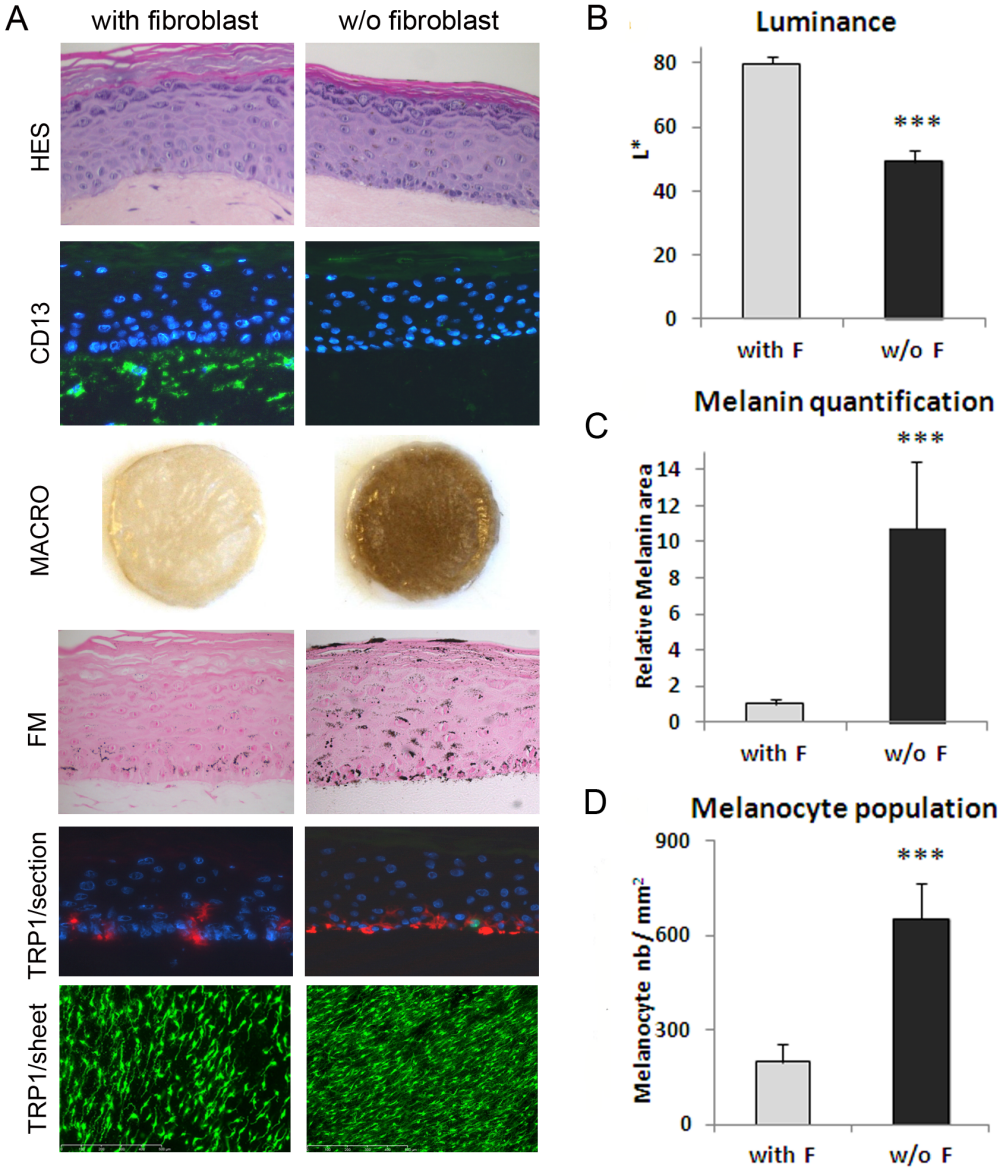
Microscopic examination and measurement of pigmentation in the *in vitro* pigmented skin model reconstructed in the presence or absence of fibroblasts in the dermal compartment. **A**) Microscopic examination of HES staining of cross-sections showed a correct epidermal architecture and differentiation process leading to the development of a stratum granulosum and stratum corneum in both 3D systems. The CD13 staining (green) of cross-sections confirmed the presence or absence of fibroblasts (from adult 21 yr-old donor) in the dermal equivalent. Macroscopic pictures of PRS illustrate the resulting drastic difference in pigmentation between the two conditions which was confirmed by **B**) Luminance measurement (mean of 3 experiments). Fontana-Masson staining for melanin granules detection (black points), and **C**) graph of melanin quantification (mean of 3 experiments) performed by image analysis of Fontana-Masson (FM) staining on cross-sections. To compare the two conditions, results on melanin content were normalized to the control condition (with fibroblasts). TRP-1 staining (red) of cross-sections shows melanocytes positioned at the basal layer of the epidermis in both 3D systems. TRP-1 staining (green) of PRS epidermal sheets and **D**) corresponding graph of melanocyte quantification (mean of 3 experiments) performed by image analysis. The values represent means ± standard deviation (SD). Statistical significance was evaluated by the Student's *t*-test; *** p-value <0.001. Magnification: A HES, FM  =  x400, CD13, TRP1/section  =  x200, TRP1/sheet  =  x50.

Melanin pigments revealed by Fontana Masson staining on 5 µm paraffin skin sections were quantified by image analysis using Histolab software (Microvision instruments, Evry, France). After determination of a threshold of detection of melanin granules, melanin content in the epidermis was quantified as the area occupied by melanin pigment within the cross-section. Melanin content was measured over the epidermal surface, defined by a standardized length (i. e. 6 mm) of dermal epidermal junction. In Fig. 1, results from the model without fibroblast are expressed relative to those obtained with the model with living fibroblasts. In other figures, melanin content is expressed as the percentage of melanin surface per epidermis surface.

### Total RNA extraction and quantitative reverse transcription PCR

Reconstructed skin sample was rinsed in Dulbecco's PBS (Invitrogen, Cergy-Pontoise, France). The epidermis and dermal equivalent were separated using fine forceps as previously described [50]. Disruption of epidermal tissue, total RNA extraction and Dnase I treatment were performed using Rneasy midi-kit (Qiagen, Courtaboeuf, France). 1 µg of total RNA was used for first strand cDNA synthesis using an Advantage RT-for-PCR kit (Clontech, Saint Quentin en Yvelines, France), according to the manufacturer's instructions. Quantitative PCR was performed using the LightCycler and the Light Cycler-Fast Start DNA Master Sybr Green kit (Roche Diagnostics, Meylan, France). Normalization of data was performed using five housekeeping genes (*B2M*, *RPL13A, RPS28, RPS9*) and Genorm application.

The following oligonucleotide primers were used: Silver sense -5′ataggtgctttgctggctgt-3′ and antisense 5′ –gcaataccttttggcttcca-3′; Tyrosinase sense 5′-ctcaaagcagcatgcacaat-3′ and antisense 5′-ccatgtaggattcccggtta-3′; Trp-1 sense 5′- tctcaggttcaaggctacaaca-3′ and antisense 5′-ccatgtaggattcccggtta-3′; DCT (Dopachrome tautomerase) sense 5′- ggcaccggtaccatttgttgtgtc-3′ and antisense 5′- gtagtcatccaagctatcacagacag- 3-; Ribosomal protein S28 sense 5′-ccgtgtgcagcctatcaag-3′ and antisense 5′- caagctcagcgcaacctc-3′; Beta-2-microglobulin sense 5′- tttcatccatccgacattga-3′ and antisense 5′- cctccatgatgctgcttaca-3′; Ribosomal protein L13a sense 5′- taaacaggtactgctgggccggaaggtg-3′ and antisense 5′- cacgttcttctcggcctgtttccgtagc-3′; Ribosomal protein S9 sense 5′- gatgagaaggacccacggcgtctgttcg-3′ and antisense 5′- gagacaatccagcagcccaggagggac-3′.

### Statistical Analysis

All experimental results are reported as mean ± standard deviation (n = 3). The p-values generated after a two-tailed Student's *t*-test were used to compare experimental values to the control. The threshold for statistical significance was 0.05 (*p<0.05, **p<0.01, ***p<0.001).

## Results

To demonstrate that the 3D skin model is capable of detecting the role of fibroblasts in the pigmentation process, we studied the effects of drastically different fibroblast conditions in the dermal equivalents. For this, two experimental approaches were designed using the pigmented reconstructed skin model (PRS): one consisted in producing PRS with or without living fibroblasts, and the second in producing PRS with fibroblasts from either fetal or adult skin. The epidermis was reconstructed with the same strains of keratinocytes and melanocytes for all models. The impact of modifying the fibroblast component on the level of pigmentation and melanocyte biomarkers was investigated.

### Influence of the presence versus absence of fibroblasts on pigmentation in PRS model

To highlight the global role of normal fibroblasts on the levels of pigmentation, reconstructed pigmented skins were produced with or without fibroblasts in the dermal compartment (Fig. 1). All analyses were performed after a culture period of 7 days in immersion phase plus 11 days in emersion phase. HES staining showed a correct morphology for both conditions and CD13 immunostaining confirmed the presence or absence of human fibroblasts in the dermal equivalent (Fig. 1A). Macroscopically, the 2 models exhibited obvious and significant differences. The model without fibroblasts was much darker as confirmed by a great decrease of luminance (ΔL  = 30, p<0.001) (Fig. 1B) and a ten-fold significant increase (p<0.001) in melanin content assessed by quantification of Fontana-Masson staining (Fig. 1C). Immunostaining of TRP1 on reconstructed skin cross-sections demonstrated the correct positioning of the melanocytes at the basal layer of the epidermis. TRP1 staining of the epidermal sheets (Fig. 1E) revealed that both models displayed homogeneous melanocyte distribution, with a correct dendritic morphology. The number of melanocytes appeared higher in the model without fibroblasts as confirmed by melanocyte quantification by image analysis (Fig. 1D). The absence of fibroblasts led to a significant increase in the mean melanocyte numbers by a factor of 3.3, while the mean melanin content increased by a factor of 10. Hence, the pigmentation-enhancing effect of dermal equivalent without fibroblasts does not seem to be linked in a linear manner to the increase in number of melanocytes.

### Influence of fibroblasts from fetal versus adult skin on pigmentation in PRS model

**Figure 2:**
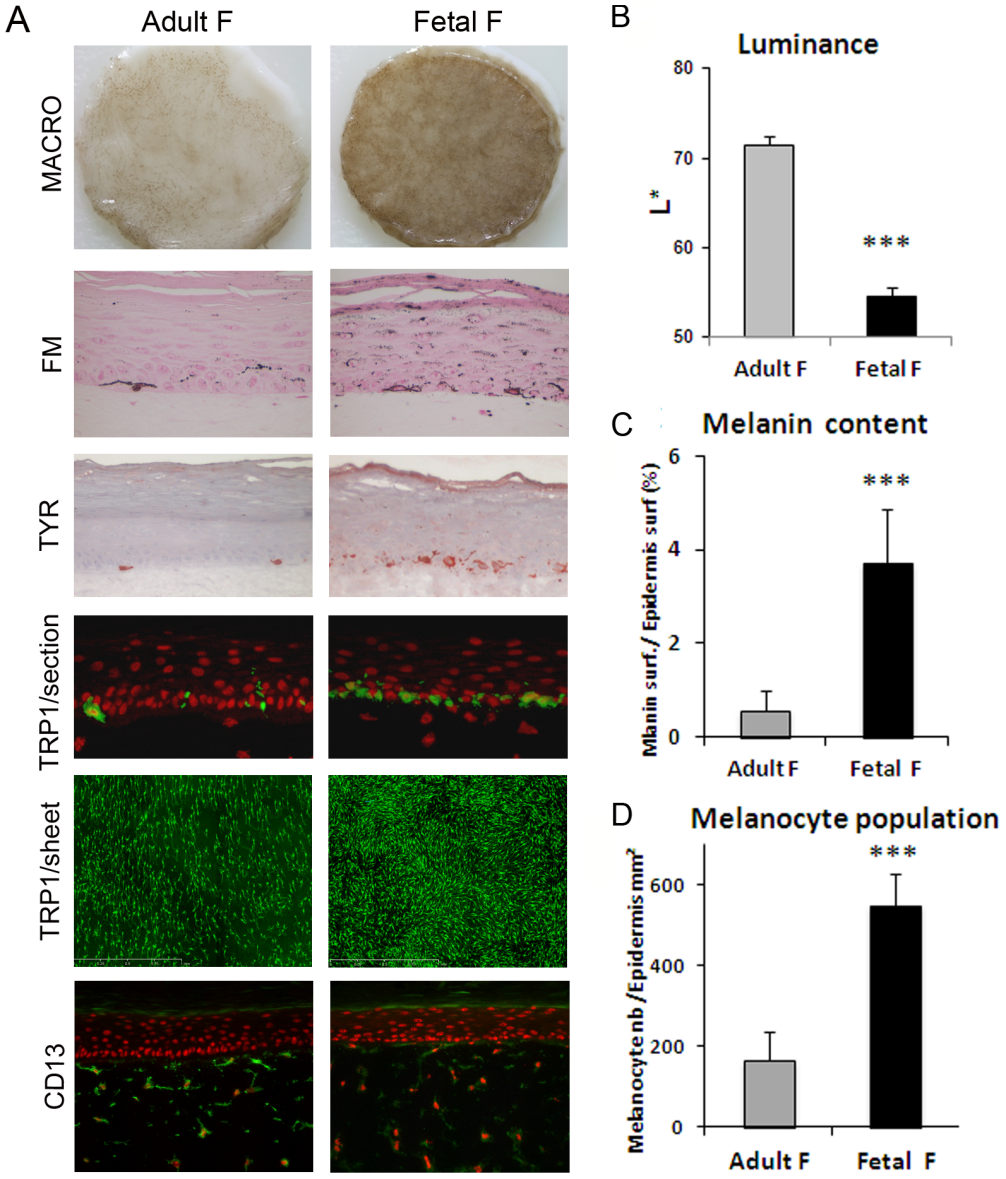
Comparison of the effect of fibroblasts from fetal versus adult origin on reconstructed skin pigmentation. PRS samples were reconstructed with either fetal fibroblasts (GM10) or adult (21 yr-old donor) fibroblasts within the dermal equivalent. Identical keratinocyte and melanocyte strains were used for the epidermal reconstruction. A drastic increase in pigmentation of the PRS and activation of melanocytes in the presence of fetal fibroblasts as compared to adult fibroblasts, were noted A**)** macroscopically (Macro), on histological sections stained with Fontana-Masson (FM) and by tyrosinase staining (Tyr) of tissue sections. The hyper-pigmentation observed in fetal versus adult fibroblast condition was quantified by **B**) a decrease in Luminance value and **C**) an increase in melanin content. TRP-1 labeling of tissue sections showed that melanocytes were correctly located at the basal layer in both conditions (**A**). TRP-1 staining of epidermal sheets revealed an increase in melanocyte numbers in the presence of fetal fibroblasts (**A**) which was confirmed by image analysis (**D**). CD13 staining revealed no change in fibroblast morphology or density (**A**). Values are expressed as the mean +/- SD calculated for 4 different samples in 2 independent experiments and analyzed using the two-tailed unpaired Student's t-test, *** p<0.001. Magnifications (**A**): FM, TYR, and TRP-1/section  =  x400, CD13/section =  x200, TRP1/sheet =  x50.

Next, in order to analyze the effect of the origin of fibroblasts on pigmentation, we compared two types of fibroblasts, either of fetal or adult origin, used at the same passage and same density. It is known that fetal and adult fibroblasts show different intrinsic properties notably regarding their capacity to repair wounds [41], [42]. An epidermis was cultured on dermal equivalents containing these two different sources of fibroblasts and drastic differences in term of pigmentation were observed. An intense pigmentation appeared, macroscopically and on histological sections, in the epidermis reconstructed on dermal equivalent with fetal fibroblasts (Fig. 2A). The hyperpigmentation of the samples prepared with fetal fibroblasts was also quantified by a decrease in luminance value (ΔL  = 16, p<0.001) and a high and significant increase in melanin content by a factor of 6.6 (p<0.001) (Fig. 2B,C). TRP-1 and tyrosinase (TYR) immunostaining of skin sections revealed that melanocytes were correctly located at the basal layer of the epidermis in both conditions, and that TYR expression was higher in the samples containing fetal fibroblasts (Fig. 2A). TRP1 staining performed on epidermal sheets clearly showed that the number of melanocytes increased in the tissues with fetal fibroblasts as compared to adult fibroblast-containing samples (Fig. 2A). This was confirmed by image analysis revealing a 3.3 fold higher population of melanocytes in the fetal condition (p<0.001) (Fig. 2D). Meanwhile, we noticed that the increase in melanin content was two-fold higher than the increase in melanocyte population in PRS samples with fetal fibroblasts. No change was noticed in terms of fibroblast morphology or distribution (CD13 staining) within the dermal equivalents (Fig. 2A).

### Influence of fibroblasts from photo-aged versus young skin on pigmentation in the PRS model

**Figure 3:**
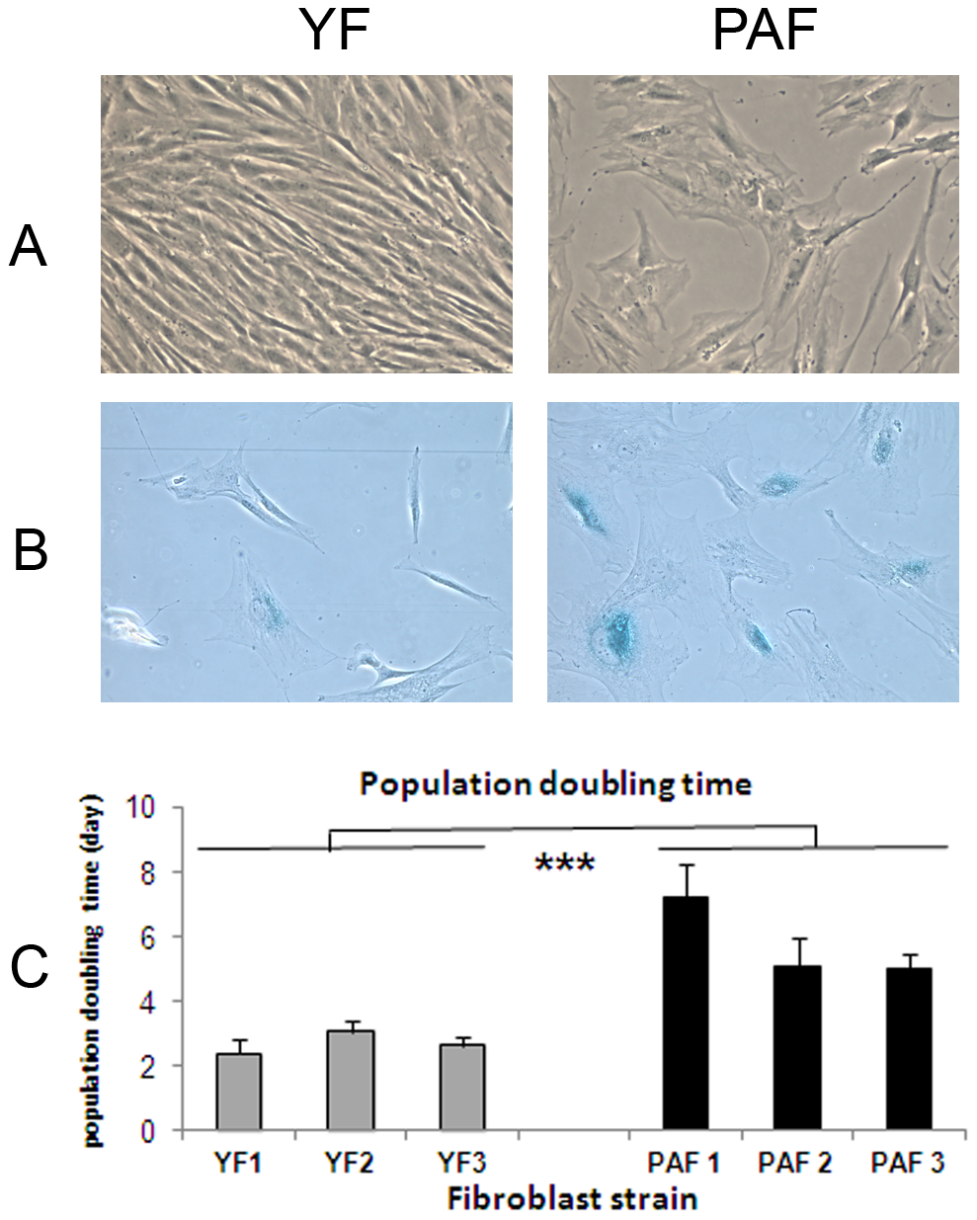
Morphology and senescence-associated β-galactosidase staining of fibroblasts from photo-aged and young skins. Fibroblasts isolated from photo-aged skin **(PAF)** and from young unexposed skin **(YF)** were grown in DMEM + 0 % FBS at 10% CO2 and passaged until P4. As shown by **A**) classical microscopy, **B**) senescence-associated β-galactosidase (SA-β-gal) staining and **C**) population doubling times (time required for a two-fold increase in fibroblast number), PAF exhibited a flattened and enlarged cellular body, a reduced growth rate and an increase in SA-β-galactosidase activity, thus revealing a senescent phenotype. Values are expressed as the mean +/- SD calculated in 3 independent experiments and analyzed using the two-tailed unpaired Student's t-test; *** p<0.001. **A** and **B** magnification  =  x200.

Our next step aimed at investigating the impact of fibroblasts coming from different real life skin types and, in particular, natural photo-aged fibroblasts, on pigmentation. The influence of fibroblasts from *in situ* photo-aged skin was compared to that of fibroblasts from young skin. For this purpose, three strains of fibroblasts isolated from photo-exposed skin of aged Caucasian donors (PAF1, PAF2, PAF3) and three strains of fibroblasts from young Caucasian donors (YF1, YF2, YF3) were amplified simultaneously to P4 in the same culture conditions. During the amplification phase in monolayers, differences in morphology and growth were noticed (Fig. 3). Compared to fibroblasts from young skin, fibroblasts from photo-aged skin exhibited a flattened cellular body, an enlarged cytoplasm and a reduced growth rate as shown by the significantly increased Population Doubling Time. Additionally, SA-β-galactosidase staining was increased in the PAF cultures. All together, these characteristics demonstrated the senescent phenotype of the PAF strains.

**Figure 4:**
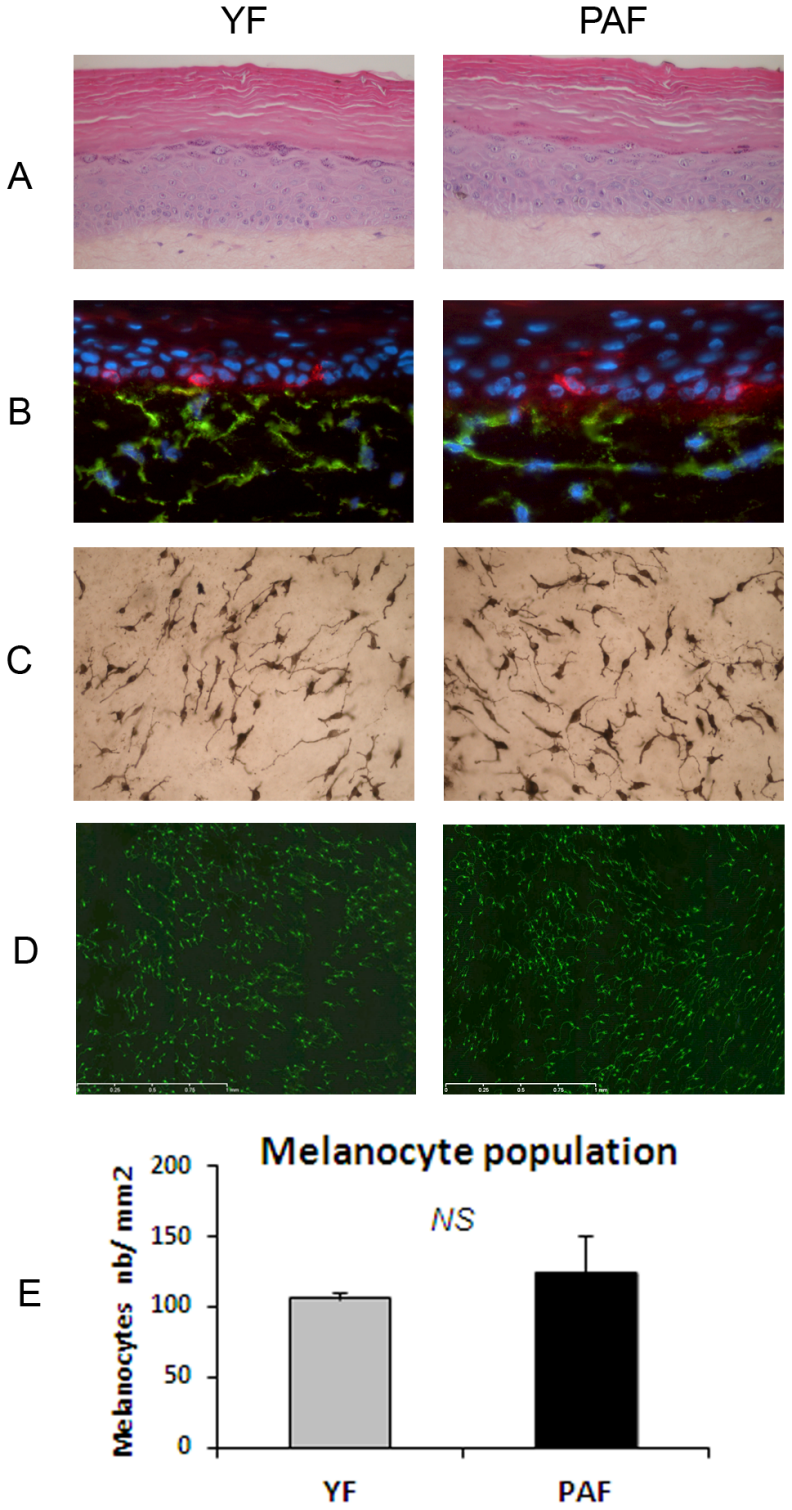
Reconstruction of pigmented skin with fibroblasts from photo-aged or young skin. Pigmented skins reconstructed with a dermal equivalent containing either photo-aged fibroblasts (PAF) or young fibroblasts (YF) were analyzed after 14 days of emersion. The same keratinocyte and melanocyte strains were used for epidermal reconstruction. In both photo-aged and young fibroblast conditions, HES colorations showed that the histological quality of the epidermis was correct (**A**). TRP-1 (red) and CD13 (green) co-staining of skin sections indicated the correct location of melanocytes in the basal layer and the presence of fibroblasts within the dermal equivalent (**B**) and Dopa reaction (**C**) and TRP1 staining (**D**) of epidermal sheets revealed the correct morphology of melanocytes in both conditions. **E**) Graph reporting the number of melanocytes as estimated by counting TRP1-positive cells/mm^2^ epidermal surface shows no significant difference between the number of melanocytes in the photo-aged versus young fibroblast condition. Values are expressed as the mean +/- SD calculated for 6 samples and analyzed using the two-tailed unpaired Student's t-test; NS: non-significant. Magnification: **A, B** =  x 400, **C** =  x200, **D** =  x50.

The 6 strains of fibroblasts were used individually at same passage and same density, to produce dermal equivalents. The epidermal cultures on these different dermal equivalents were prepared using the same strains of NHK and NHM. The histological features of the reconstructed epidermis, especially keratinocyte differentiation, were correct in all cases (Fig. 4A). Fibroblasts were well-integrated within the dermal matrix while they displayed an elongated shape (Fig. 4B), a feature previously described in fibroblasts from photo-aged skin [51].

Melanocyte behavior and pigmentation in the PAF versus YF conditions were compared. Melanocytes showed a correct location at the DEJ, a dendritic morphology and a regular distribution within the epidermis in both conditions (Fig. 4B–D). Quantification of melanocytes on the epidermal sheets revealed no significant difference in melanocyte numbers in the reconstructed skin samples containing photo-aged fibroblasts as compared to those with young fibroblasts (Fig. 4E).

**Figure 5:**
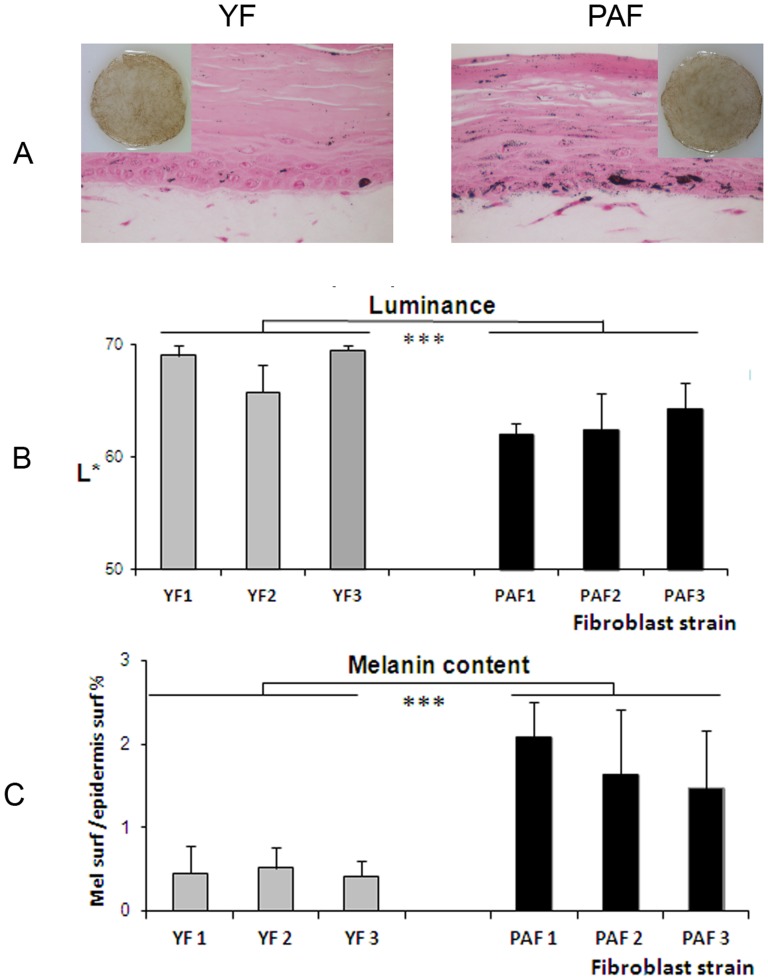
Pigmentation levels in skin reconstructed with natural photo-aged fibroblasts versus young fibroblasts. Pigmentation in the skin reconstructed with either natural photo-aged fibroblasts (PAF) or young fibroblasts (YF) was analyzed macroscopically and on histological sections stained with Fontana Masson (**A**) and quantified by Luminance measurements (L*) (**B**) and melanin quantification (**C**) after 14 days of emersion. In the presence of the three PAF strains versus the three YF conditions, a significant increase in pigmentation was observed as shown by i) an increase in the concentration of melanin granules as measured by image analysis on histological sections, and ii) the darkening of the skin samples which correlates with a decrease in Luminance. Values are expressed as a mean +/- SD calculated for 3 samples in 3 independent experiments and analyzed using the two-tailed unpaired Student's *t*-test; *** p<0.001. Magnification: **A** =  x400.

**Figure 6:**
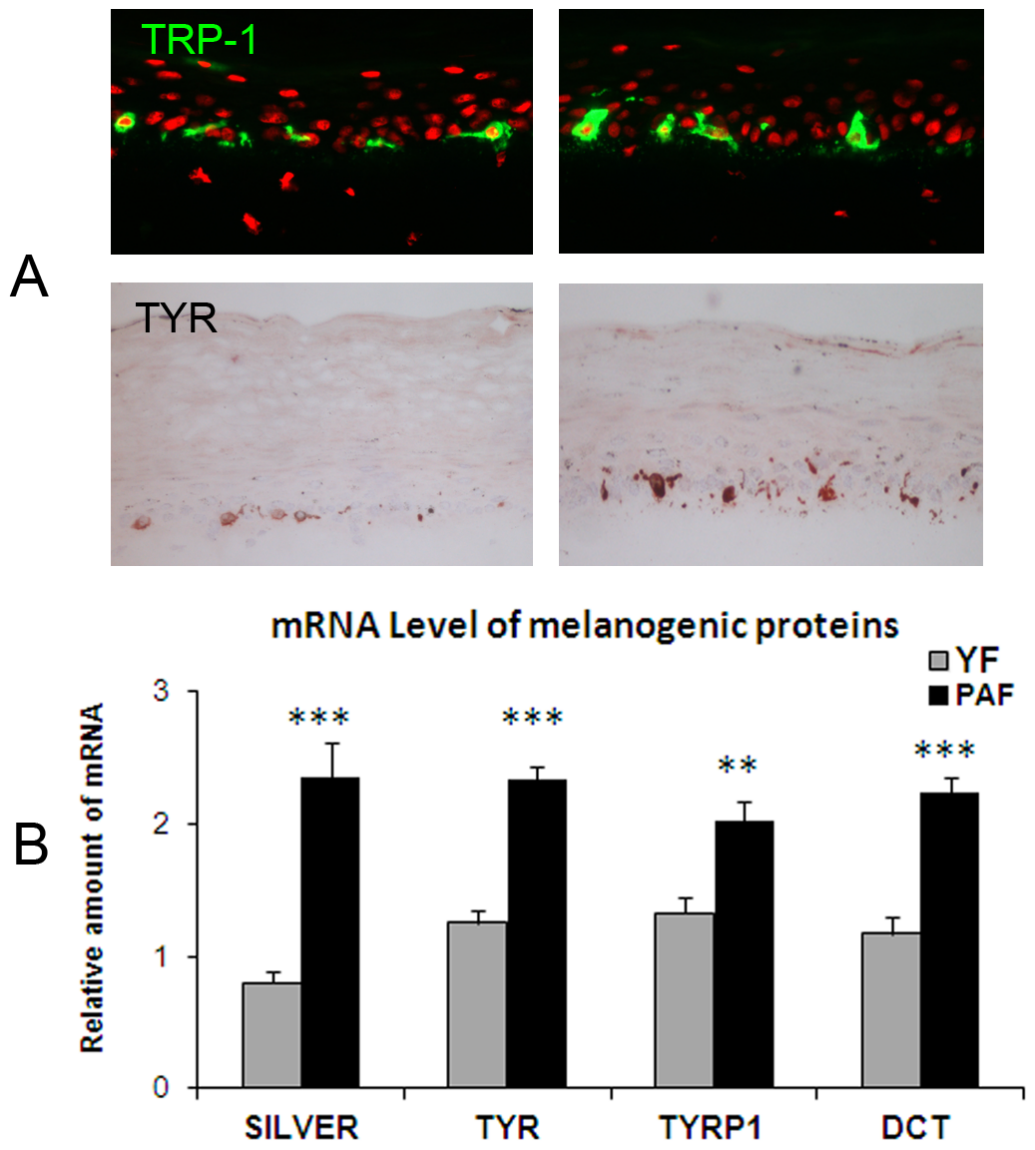
Effect of fibroblasts from photo-aged versus young skin on the expression level of melanogenic proteins in reconstructed skin. Analysis of melanocyte location and activity in the reconstructed skin samples containing either photo-aged fibroblasts (PAF) or young fibroblasts (YF) was analyzed by immunostaining on histological sections using TRP-1 and Tyrosinase antibodies (**A**) and by measuring expression levels of melanogenic genes by RT-qPCR (**B**). Immunostaining of tissue sections showed the correct location of melanocytes in the basal layer and increased labeling of Tyrosinase in PAF conditions as compared to YF samples. The analysis of expression levels of genes involved in melanogenesis in the epidermis revealed the increase in mRNA levels of Silver, Tyrosinase, TRP-1 and DCT (TRP-2) in the PAF-containing model as compared to the YF condition confirming that melanocytes were stimulated when photo-aged fibroblasts were present in dermal equivalent. Values are expressed as a mean +/- SD calculated for 3 samples in one experiment and analyzed using the two-tailed unpaired Student's *t*-test; ** p<0.01, *** p<0.001. Similar results were obtained in three independent experiments. Magnification: A =  x400.

Interestingly, a higher level of pigmentation was observed in the tissues with natural photo-aged versus young fibroblasts. In the presence of the three PAF strains versus the YF conditions, a significant increase in pigmentation of the skin model was induced as demonstrated by i) a higher amount of melanin granules as seen on histological sections and measured by image analysis, and ii) the darkening of the skin samples as quantified by a decrease in luminance (Fig. 5). Additionally, TRP1 and TYR immunostainings of skin sections revealed that melanocytes were correctly located at the basal layer and activated as shown by intense enzyme staining (Fig. 6A). These results were strengthened by the analysis of expression of melanogenesis genes in the epidermis revealing an increase in mRNA levels of Silver, Tyrosinase, TRP-1 and DCT (TRP-2) in the presence of PAF versus YF (Fig. 6B).

To address the question of the role of fibroblast-derived soluble factors on the hyperpigmented phenotype, we selected from the literature 18 factors (cytokines, chemokines, growth factors) known to modulate pigmentation, even though they have not been specifically associated with a fibroblastic origin. These factors are listed in S1 Figure. Culture supernatants of dermal equivalents prepared with the six strains of young photo-protected or naturally photo-aged fibroblasts were collected after the dermal contraction phase. Using the ELISA technique, the concentration of the 18 soluble factors was measured. Only GM-CSF was found to be significantly different between young photo-protected and photo-aged fibroblast conditions with lower amounts observed in the presence of the photo-aged fibroblasts (S1 Figure). In photo-aged fibroblasts dermal equivalents, SCF levels appeared to increase but not in a significant manner (p = 0.085) while αMSH amounts appeared to decrease but, again, the result was not significant (p = 0.086). ET-1 and Wnt-3a were not detected and the other tested factors (LIF, NRG1-b1, b-NGF, HGF, BMP-4, IL-6, b-FGF, DKK-1, Il-1α, MIF, MCP-1, FGF-7, ET-3) did not show differences between the two types of samples. Examination of the levels of the factors produced in the different conditions revealed an inter-strain variability even more pronounced in the photo-aged fibroblasts strains. Examples of data obtained for each strain and showing this variability are illustrated in S1 Figure.

## Discussion

In order to study cutaneous pigmentation in a physiological context, we have developed a pigmented reconstructed skin which reproduces i) the 3-D architecture of the melanocyte environment, ii) the interactions between melanocytes and their active cellular partners, keratinocytes and fibroblasts, iii) the functionality of the pigmentary system [40]. One of the advantages of this model is the possibility to vary the source of the epidermal cells (melanocytes or keratinocytes) or the dermal cells independently. Thus, analysis of interactions between the different cells types is possible and the role of each on skin pigmentation can be individually evaluated. Particularly, this skin model constitutes a unique tool to study the global impact of fibroblasts on skin pigmentation. This can occur through multiple mechanisms and, in particular, through the secretion of soluble factors or the production of various extracellular matrix proteins that make up the epidermal-dermal junction. The PRS used here is also the only *in vitro* model that allows macroscopic observation of skin pigmentation changes. The purpose of this study was to highlight the influence of fibroblasts on pigmentation in the PRS model by modifying only the fibroblastic component within the dermal equivalent and to prove that the nature/origin or the cell history can have a significant impact on this phenomenon.

First experiments were designed to validate the responsiveness of the model by modifying its dermal fibroblastic component to find out if this results in pigmentation modulations.

Pigmented reconstructed skins were first produced in the presence or absence of fibroblasts in the dermal compartment in order to examine the global impact of normal fibroblasts upon pigmentation. Our results showed an intense increase in the pigmentation level associated with a higher melanocyte pool in the fibroblast-deprived skin model. These data indicated that the presence of dermal cells restrains the melanocyte pool and melanogenesis process. It should be noted that these effects may be indirectly mediated via keratinocytes. Our results are in line with previous data published by Hedley et al [52] and Cario-André et al [35] who performed similar experimentation on a pigmented epidermis reconstructed on an acellular de-epidermized dermis (DED). The authors showed that the level of pigmentation decreased when the DED was recolonized by fibroblasts. However, in this experimental system, the re-colonization process could not be accurately controlled in terms of fibroblast density and localization in the dermal support. Consequently the comparison between different strains of fibroblasts can be hardly achieved. The interest of the 3D skin model presented in this work is that the system offers a more physiological approach with regards to distribution and behavior of dermal fibroblasts and also exhibits greater dynamics in terms of pigmentation modulation.

In contrast to the work performed on 3D models, a study using 2D melanocyte cultures revealed that conditioned medium from fibroblastic monolayers played a stimulating role on the pigmenting activity and the size of melanocyte pool [53]. This contradictory result is probably due to the limited physiological relevance of the 2D mono-cellular model.

Some fibroblastic soluble factors have been identified as inhibitors of the pigmentary function, especially Dickkopf 1 (DKK1) which is expressed at high levels in fibroblasts originating from the palms and soles, and is involved in the very light pigmentation observed at these body sites [15]. A comparison, using ELISA measurements, of the culture media of the two conditions (with or without fibroblasts) was conducted to quantify concentrations of DKK1 and the related factor DKK3, both members of the Dickkopf family known as Wnt modulators. We indeed found differential DKK1 and DKK3 levels in the culture media with expression levels being higher in the lighter-pigmented, fibroblast-containing condition (S2 Figure). Our results are thus in agreement with a negative regulatory role of DKK1 and DKK3 on pigmentation and in accordance with the depigmenting effect observed when DKK1 was added to a pigmented reconstructed epidermis (lacking fibroblasts) [15].

IL6 is another soluble factor produced by fibroblasts and that has been described to have an inhibitory effect on tyrosinase activity and melanocyte proliferation [54]. In our experimental system, high concentrations of IL6 were detected when fibroblasts were present whereas the cytokine was undetectable in the condition without fibroblasts. IL6 could thus also be implicated in the hypo-pigmented phenotype of the fibroblast-containing PRS but other fibroblast-derived paracrine factors cannot be ruled out.

After completing this global demonstration of the impact of dermal fibroblasts on pigmentation, we evaluated the influence of fibroblast origin on the same endpoints. Interestingly, we observed a drastic increase in pigmentation of skin reconstructed on a dermal equivalent with fetal fibroblasts as compared to that with adult fibroblasts. These results show that the skin model is indeed responsive to changes in the nature of the fibroblast and suggest that intrinsic differences between the two types of fibroblasts could trigger variation in pigmentation. It is known that fetal fibroblasts are responsible for scar-free wound repair and rapid healing [41], [42]. These cells express different gene and protein profiles related to ECM production and also secrete different growth factors and inflammatory cytokines as compared to adult or neonatal fibroblasts [55]–[57]. In this work, we collected the supernatants of dermal equivalents seeded with either fetal or adult fibroblasts and quantified a certain number of factors previously shown to be produced by fetal fibroblasts and also known for their effects on melanocytes or melanogenesis. Of particular interest, the work of Pouyani et al [56] reported the increase in NRG-1 and HGF gene expression in fetal fibroblasts embedded in dermal constructs. Although NRG-1 was not detected in any of our conditions, HGF was detected and was present in higher amounts in the supernatant of dermal equivalents with fetal fibroblasts (S2 Figure). HGF is largely described as a mitogenic factor for melanocytes [58], [59] and may therefore contribute to the increase in melanocyte numbers observed in the fetal fibroblast condition. We also tested DKK1 but did not detect a differential expression. Among factors highly expressed in fetal skin and involved in the wound healing process, Macrophage Migration Inhibitory Factor (MIF) has been reported to promote an increase in the pigmentation of reconstructed epidermis probably through keratinocyte mediation and SCF release [60], [61]. MIF levels were assessed in our experimental conditions and, indeed, found to be highly secreted in dermal equivalents with fetal fibroblasts as compared to those prepared with adult cells. In summary, MIF could play a role, in combination with HGF, in the hyper-pigmented phenotype of the PRS model containing fetal fibroblasts.

This experimentation allowed us to establish a proof of principle attesting that dermal fibroblasts do indeed impact skin pigmentation and that the model used in our laboratory is adequate for detecting such impacts. To further study the influence of fibroblasts in conditions that may be more pertinent to human adult skin, we decided to work with fibroblasts isolated from photo-aged skin. Photo-aged skin is characterized by mottled, irregular areas of pigmentation and hyper-pigmented lesions such as actinic lentigines in sun-exposed areas [27], [30], [32]. It is well known that skin photo-aging is also associated with profound alterations of the dermis and dermal fibroblasts [43], [51], [62], [63]. To investigate the influence of chronically photo-exposed fibroblasts on pigmentation, we compared epidermis reconstructed on dermal equivalents containing fibroblasts from photo-aged skin (3 different strains, donors aged over 70 yrs of age) with reconstructed skin samples containing fibroblasts from young, unexposed skin (16–21 yrs old, 3 strains). Prior to fibroblast integration into the dermal equivalent, we confirmed that natural photo-aged fibroblasts displayed a senescent profile with a lower proliferation rate, altered morphological features and the β-galactosidase expression [64].

Our data from the 3D models demonstrate that the presence of natural photo-aged fibroblasts, as compared to young unexposed fibroblasts, within the dermal equivalent stimulates epidermal pigmentation as revealed by a decrease in luminance value, an increase in melanin content and an increase in melanogenic genes expression. In addition, these effects were obtained without any significant modification of melanocyte numbers. To our knowledge, this is the first *in vitro* demonstration, from visual assessment to gene expression levels, of a direct link between dermal photo-aging and pigmentation.

Previous experimental approaches using stress–induced premature senescent (SIPS) fibroblasts generated by treatment with PUVA [65], UVA or UVB [66] identified cytokines released by SIPS-fibroblasts. The presence of known pro-melanogenic cytokines, such as SCF, KGF and HGF was outlined. However, their involvement in photo-aged hyperpigmented phenotype is not demonstrated. In addition, SIPS-fibroblasts may only partially reflect the cellular photo-aging phenotype due to the artificial way they are produced as compared to the conditions used here in which the cells acquired a natural UV exposure history. The natural photo-aged fibroblasts we used were directly isolated from skin biopsies and clearly retained their long history of chronic sun exposure of the skin even after being isolated and cultured *in vitro.*

Another key element of the present study is the fact that photo-aged fibroblasts within the dermal equivalent do not, as *in vivo*, proliferate. This quiescent status may have important consequences in term of fibroblast metabolism and secretory profile. Kim et al [67] recently demonstrated, using a virtual skin model, that melanocyte homeostasis can be modified by disrupted stromal cells, namely, artificially-induced senescent fibroblasts. In an attempt to address the mechanistic clues underlying our observations, we tested 18 soluble factors known to modulate pigmentation. Among those detected in the supernatants of the dermal equivalents, GM-SCF was significantly decreased in the photo-aged condition as compared to the samples with young unexposed fibroblasts. We also noted that SCF was increased and αMSH was reduced (tendency). Both GM-CSF and αMSH are well-known stimulators of melanocytes but in our study they are found decreased in the most pigmented condition. SCF, also known to activate pigmenting cells, did increase in the hyper-pigmented phenotype but this increase was not found in one of the three strains of photo-aged fibroblasts. Therefore it cannot be directly linked to the increased level of pigmentation of the corresponding PAF–containing skin model (see S1 Figure). For the others factors quantified, high variability was observed between the 3 strains used for each condition and a potential causal effect between their production and the pigmentation increase cannot therefore be hypothesized.

Further global proteomic investigations will be needed to characterize and validate more precisely the growth factors or cytokines released by the fibroblasts that could be actors in regulating pigmentation during photo-aging. It also has to be stressed that the mechanisms underlying the pigmentation phenotype could be trigger by ECM proteins which were not studied here.

In conclusion, the complex pigmented skin model allowing mesenchymal–epidermal interactions used here provides for the first time an *in vitro* macroscopic visualization of the role of dermal fibroblasts on skin pigmentation levels. The model has the advantage of permitting a global assessment of the contribution of fibroblasts which could be mediated by either soluble factors or ECM components. We have provided evidence that the presence, the origin and, more importantly, the history and acquired characteristics of dermal fibroblasts are indeed modulators of the level of pigmentation. Finally, our data suggest that fibroblasts from chronically sun-exposed skin may contribute to hyperpigmentation observed during photo-aging. Although the precise mechanisms of action have not be elucidated at the present time, the tools employed here allow us to envisage further investigations on ECM proteins and soluble factors in order to unravel the underlying molecular mechanisms.

## Supporting Information

S1 Figure **ELISA analysis of secreted soluble factors from culture supernatants of dermal equivalents containing fibroblasts from either photo-aged or young unexposed skin.** Culture supernatants of dermal equivalents containing natural photo-aged fibroblasts (3 strains, Passage 4) or young unexposed fibroblasts (3 strains, passage 4) was harvested after the contraction phase (4 days after the mixing of fibroblasts and collagen). Soluble factors (cytokines, chemokines, growth factors) known to modulate melanogenesis, even not specifically associated with a fibroblastic origin, were measured by the Elisa technique. The results in the table are expressed as the mean +/- standard deviation for each condition and analyzed using the two-tailed unpaired Student's *t*-test (* p<0.05). Ten illustrative graphs show the quantity of factor found for each individual fibroblast strains.Among the tested factors, only GM-SCF was found to be significantly decreased in the supernatant of photo-aged fibroblast-containing dermal equivalent as compared to the young, unexposed fibroblast condition. Modulation of SCF (increase) and MSH (decrease) shows a tendency but without reaching significance (p =  0.085 and p = 0.086 respectively). Two factors, ET-1 and Wnt-3a, were not detected (nd). Regarding the others factors, important variations between the 3 strains used for each condition (and especially the photo-aged fibroblast strains) was revealed by the high standard deviation values and the wide repartition of the values on the graphs.
(TIF)

S2 Figure **ELISA analysis of secreted soluble factors from culture supernatants of studied models.** **A**) Pigmented reconstructed skin with or without fibroblasts, **B**) dermal equivalents containing fetal fibroblasts or adult fibroblasts. Values indicate cytokine/growth factors concentrations in pg/ml. Nd, not detectable. Two tailed Student's t-test * p<0.5, ** p<0.01. Following factors known to be involved in the pigmentation regulation have been tested in both models: b-FGF, KGF, SCF, IL1α, IL6, BMP6, BMP4, DKK1, DKK3, Wnt3a, Wnt5a, MIF, NRG1-b1. Apart from the graphs shown in this Figure, the measurements resulted either in undetectable or not significantly modulated levels of the tested soluble factors.
(TIF)
