# Supplementary figures and images for: Key Regulatory Role of Dermal Fibroblasts in Pigmentation as Demonstrated Using a Reconstructed Skin Model: Impact of Photo-Aging
Source: PLoS One. 2014 Dec 9;9(12):e114182. doi: 10.1371/journal.pone.0114182 (PMC4260844; doi:10.1371/journal.pone.0114182)

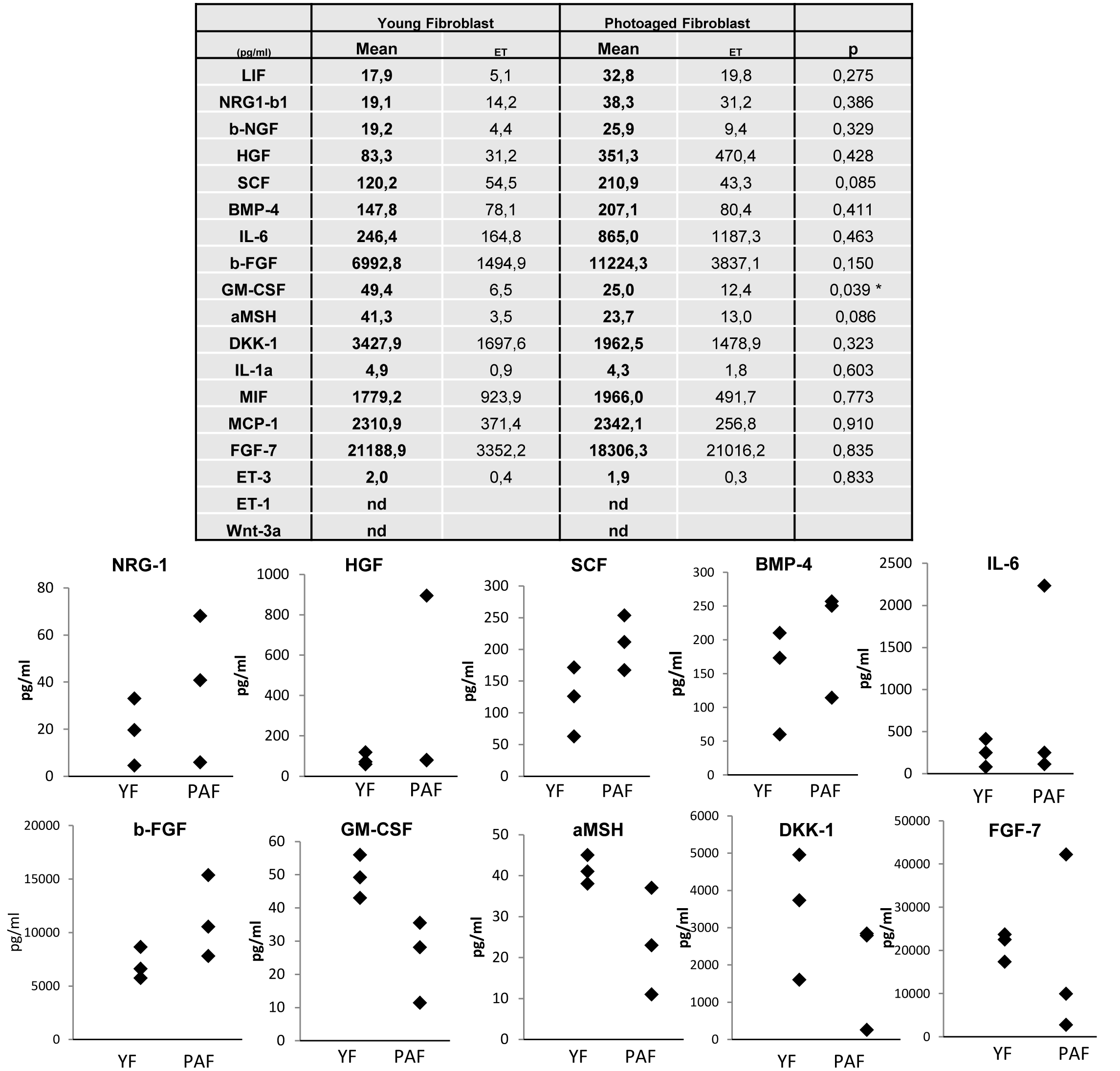

Supplement: S1 Figure — ELISA analysis of secreted soluble factors from culture supernatants of dermal equivalents containing fibroblasts from either photo-aged or young unexposed skin. Culture supernatants of dermal equivalents containing natural photo-aged fibroblasts (3 strains, Passage 4) or young unexposed fibroblasts (3 strains, passage 4) was harvested after the contraction phase (4 days after the mixing of fibroblasts and collagen). Soluble factors (cytokines, chemokines, growth factors) known to modulate melanogenesis, even not specifically associated with a fibroblastic origin, were measured by the Elisa technique. The results in the table are expressed as the mean +/- standard deviation for each condition and analyzed using the two-tailed unpaired Student's t-test (* p<0.05). Ten illustrative graphs show the quantity of factor found for each individual fibroblast strains.Among the tested factors, only GM-SCF was found to be significantly decreased in the supernatant of photo-aged fibroblast-containing dermal equivalent as compared to the young, unexposed fibroblast condition. Modulation of SCF (increase) and MSH (decrease) shows a tendency but without reaching significance (p = 0.085 and p = 0.086 respectively). Two factors, ET-1 and Wnt-3a, were not detected (nd). Regarding the others factors, important variations between the 3 strains used for each condition (and especially the photo-aged fibroblast strains) was revealed by the high standard deviation values and the wide repartition of the values on the graphs. (TIF) [file pone.0114182.s001.tif]

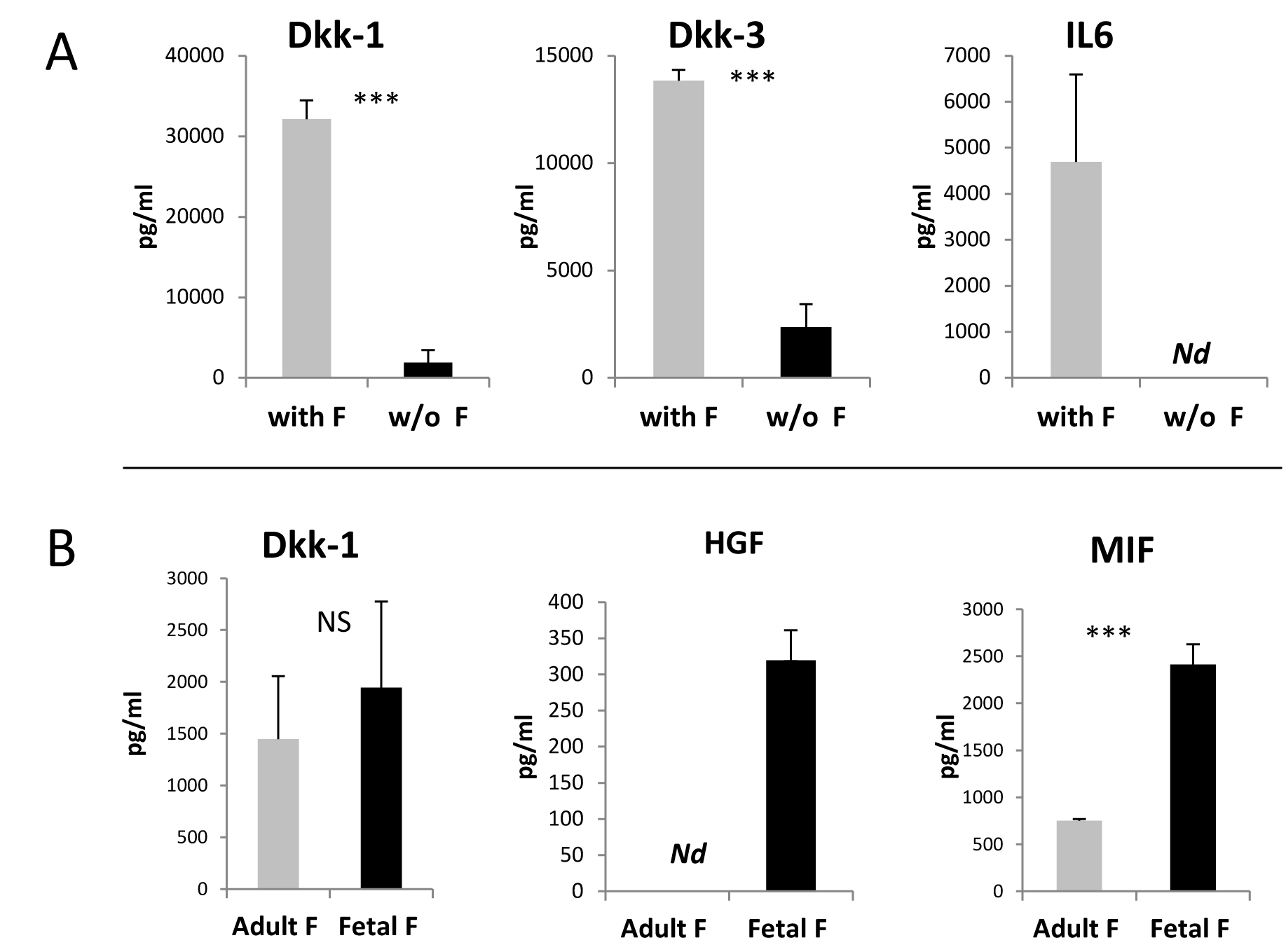

Supplement: S2 Figure — ELISA analysis of secreted soluble factors from culture supernatants of studied models. A) Pigmented reconstructed skin with or without fibroblasts, B) dermal equivalents containing fetal fibroblasts or adult fibroblasts. Values indicate cytokine/growth factors concentrations in pg/ml. Nd, not detectable. Two tailed Student's t-test * p<0.5, ** p<0.01. Following factors known to be involved in the pigmentation regulation have been tested in both models: b-FGF, KGF, SCF, IL1α, IL6, BMP6, BMP4, DKK1, DKK3, Wnt3a, Wnt5a, MIF, NRG1-b1. Apart from the graphs shown in this Figure, the measurements resulted either in undetectable or not significantly modulated levels of the tested soluble factors. (TIF) [file pone.0114182.s002.tif]
